# Peroxide of Hydrogen for the Relief of Bites from Venomous Insects

**Published:** 1890-03

**Authors:** 


### PEROXIDE OF HYDROGEN FOR THE RELIEF OF BITES FROM VENOMOUS INSECTS.

Dr. Phillipe Ricord of Newark, N. J., writes: "Recently, while charging my atomizer with the full strength of frest standard Marchard preparation of peroxide of hydrogen, at the bedside of a child suffering with diphtheria, my attention was attracted by the patient's mother, who appeared in pain and stated that, while taking up a blanket to wrap about her child, she supposed she had been pricked by a needle, and, on further examination, discovered a hornet between the folds she had touched. Thereupon I immediately directed the peroxide of hydrogen spray into the wound, the surrounding tissues, in the few seconds that had elapsed, being swollen to such an extent as to distinctly mark its site. Instantly all pain ceased, and the swelling rapidly disappeared. In this case the wound was still sufficiently open to readily admit the peroxide of hydrogen, and the destruction of the virus was, apparently, in a moment so completely accomplished that no further treatment was afterward required. May we not, therefore, infer that it is quite possible to annihilate many other poisons likewise, by the prompt application of so powerful, yet safe, an agent as the peroxide of hydrogen?"—*Medical Record.*

